# Nucleotide synthesis is regulated by cytoophidium formation during neurodevelopment and adaptive metabolism

**DOI:** 10.1242/bio.201410165

**Published:** 2014-10-17

**Authors:** Gabriel N. Aughey, Stuart J. Grice, Qing-Ji Shen, Yichi Xu, Chia-Chun Chang, Ghows Azzam, Pei-Yu Wang, Luke Freeman-Mills, Li-Mei Pai, Li-Ying Sung, Jun Yan, Ji-Long Liu

**Affiliations:** 1 Medical Research Council Functional Genomics Unit, Department of Physiology, Anatomy and Genetics, University of Oxford, Oxford OX1 3PT, United Kingdom; 2 CAS-MPG Partner Institute for Computational Biology, Shanghai Institutes of Biological Sciences, Chinese Academy of Sciences, Shanghai 200031, China; 3 Institute of Biotechnology, National Taiwan University, Taipei 106, Taiwan, Republic of China; 4 Department of Biochemistry, College of Medicine, Chang Gung University, Tao-Yuan, 333, Taiwan, Republic of China; 5 Molecular Medicine Research Center, College of Medicine, Chang Gung University, Tao-Yuan 333, Taiwan, Republic of China; 6 Graduate Institute of Biomedical Sciences, College of Medicine, Chang Gung University, Tao-Yuan 333, Taiwan, Republic of China; 7 Agricultural Biotechnology Research Center, Academia Sinica, Taipei 115, Taiwan, Republic of China

**Keywords:** CTP synthase, cytoophidium, intracellular compartmentation, CTP, *Drosophila*, neurogenesis

## Abstract

The essential metabolic enzyme CTP synthase (CTPsyn) can be compartmentalised to form an evolutionarily-conserved intracellular structure termed the cytoophidium. Recently, it has been demonstrated that the enzymatic activity of CTPsyn is attenuated by incorporation into cytoophidia in bacteria and yeast cells. Here we demonstrate that CTPsyn is regulated in a similar manner in *Drosophila* tissues *in vivo*. We show that cytoophidium formation occurs during nutrient deprivation in cultured cells, as well as in quiescent and starved neuroblasts of the *Drosophila* larval central nervous system. We also show that cytoophidia formation is reversible during neurogenesis, indicating that filament formation regulates pyrimidine synthesis in a normal developmental context. Furthermore, our global metabolic profiling demonstrates that *CTPsyn* overexpression does not significantly alter CTPsyn-related enzymatic activity, suggesting that cytoophidium formation facilitates metabolic stabilisation. In addition, we show that overexpression of *CTPsyn* only results in moderate increase of CTP pool in human stable cell lines. Together, our study provides experimental evidence, and a mathematical model, for the hypothesis that inactive CTPsyn is incorporated into cytoophidia.

## INTRODUCTION

In recent years it has become apparent from protein localisation screens that an unprecedented number of metabolic enzymes are incorporated into novel cytoplasmic bodies (Narayanaswamy et al., 2009; Werner et al., 2009). The extent to which these aggregates represent biologically meaningful structures remains unclear; however there are well characterised examples in which the assembly of higher-order structures regulates flux through specific metabolic pathways. For example, it has been observed that six of the key enzymes involved in the *de novo* synthesis of purine nucleotides reversibly colocalise to discrete cytoplasmic bodies (purinosomes) in response to depletion of purine concentrations (An et al., 2008). It is thought that this aggregation occurs to facilitate substrate channelling between enzymes in the same pathway, although recent evidence has questioned whether these bodies are physiologically relevant (Zhao et al., 2014).

A distinct subset of cytoplasmic bodies has been identified in which enzymes are incorporated into filamentous structures. One of the earliest enzymes to be identified as subject to regulation by the assembly of monomers into higher-order structures is acetyl-CoA carboxylase (ACC). Purified ACC from animal tissues has been shown by electron microscopy to reversibly form filamentous structures upon incubation with its allosteric activator, citrate (Kleinschmidt et al., 1969). Conversely, incubation with malonyl CoA or Mg^2+^ ions, both inhibitors of ACC, caused rapid depolymerisation of citrate-induced filaments, indicating that polymerisation of ACC is required to upregulate enzymatic activity (Beaty and Lane, 1983a; Beaty and Lane, 1983b). One of the best studied enzyme filaments so far is the essential *de novo* pyrimidine biosynthesis enzyme, CTP synthase (CTPsyn). CTPsyn-containing structures have been termed cytoophidia due to their apparent snake-like morphology (from the Greek *cyto*, meaning cell; and *ophidia*, meaning serpents) (Liu, 2010). The filament forming properties of CTPsyn have been shown to be highly conserved having been demonstrated in bacteria (Ingerson-Mahar et al., 2010), yeast (Noree et al., 2010), *Drosophila* (Liu, 2010) and human cells (Carcamo et al., 2011; Chen et al., 2011). The conservation of this feature throughout evolution suggests that the ability of CTPsyn to assemble into filamentous structures confers an important biological function (for a review, see Liu, 2011).

The enzymatic activity of CTPsyn is regulated by all four nucleotides, through substrate concentration/energy generation (UTP, ATP) and allosteric interactions (CTP, GTP) (Levitzki and Koshland, 1971; Levitzki et al., 1971; Lieberman, 1956; Long and Pardee, 1967). ATP, UTP and CTP all promote assembly into the tetramer form of the enzyme, and have been shown to increase catalytic activity, whilst GTP acts as a positive allosteric activator of the glutaminase activity of the enzyme, although this has been shown to be non-essential *in vitro* (Aronow and Ullman, 1987; Endrizzi et al., 2004; Goto et al., 2004; Pappas et al., 1998; Robertson, 1995). Treatment of *S. cerevisiae* with CTP, an inhibitor of CTPsyn activity, was shown to increase filament formation (Noree et al., 2010). The compartmentalisation of CTPsyn has been reported to be influenced by a variety of different extrinsic factors. Treatment of *S. cerevisiae* with the non-specific kinase inhibitor staurosporine resulted in an increased abundance of cytoophidia, leading to the suggestion that cytoophidium formation may be dependent on phosphorylation of CTPsyn itself or of a critical regulator (Noree et al., 2010). These data must be interpreted with caution however, due to the broad effects of staurosporine which are known to cause toxicity (Tamaoki et al., 1986).

Here we report that CTPsyn is compartmentalised in response to nutrient stress in cultured *Drosophila* cells. Furthermore we show that this effect is also apparent in *Drosophila* tissues *in vivo*. We also show that dispersal of CTPsyn occurs in larval neuroblasts *in vivo* upon exiting quiescence, indicating that the formation of cytoophidia occurs just prior to neuronal stem cell activation. In addition, cytoophidia formation occurs in neuroblasts when young larvae are starved – a process that stalls larval growth and cell proliferation (Tennessen and Thummel, 2011). We then aimed to further understand the metabolic effects of CTPsyn compartmentalisation. To this end, we performed metabolomic profiling of 308 metabolites on adult flies and found that overexpressing CTPsyn does not change CTPsyn-related enzymatic activity, suggesting that cytoophidium formation aids metabolic stabilisation. Furthermore, we show that overexpression of CTPsyn only results in moderate increase of CTP pools in human stable cell lines. Finally, we report that point mutations at critical residues in the tetramer and dimer interfaces of CTPsyn have an effect on cytoophidia formation indicating that CTPsyn multimers may be incorporated into cytoophidia. Based on our experimental data, we propose a simple mathematical model to explain the dynamics of cytoophidium formation, which accurately predicts CTPsyn concentration dependent changes of intracellular CTP nucleotides.

## MATERIALS AND METHODS

### Cell culture

*Drosophila* S2R+ cell lines obtained from the *Drosophila* Genome Resource Centre (DGRC) were grown at 25°C in Schneider's medium with 10% heat-inactivated fetal bovine serum (FBS) following standard protocols. Human 293T cells were maintained in DMEM (Invitrogen, 11960) supplemented with 10% FBS (Bioindustry), glutamine 2 mM (Invitrogen, 25030) and 1% Penicillin–Streptomycin solution (Gibco, 15140). Cells were kept in a 37°C incubator with 5% CO_2_. For western blot assays, transfected 293T cells were selected with puromycin 10 µg/ml (InvivoGen, ant-pr-5b).

### Transfection of *Drosophila* and human cells

*Drosophila* S2R+ cells were diluted to 1×10^6^ cells/ml and left to settle on coverslips in a 24 well plate overnight. If cells were intended for immunostaining, autoclaved coverslips were added to wells for cells to adhere to. 1 µg of DNA with 3 µl of FuGENE HD was added to 50 µl of water and mixed by flicking. DNA-FuGENE mixture was incubated at room temperature for 20 minutes. The DNA-FuGENE solution was dropped evenly onto the S2R+ cells and left to transfect for 24 hours before preparing samples for immunofluorescence. Control wells containing no plasmid DNA or empty vector were included for each transfection experiment. To generate stable cell lines, 4.5 µg expression constructs were co-transfected with 0.5 µg pCopuro puromycin resistance plasmid (Addgene). Cell culture medium was supplemented with 2 µg/ml puromycin after 48 hours to select against non-transfected cells. Cultures were maintained in 2 µl/ml puromycin thereafter.

For transfection of human 293T cells, full length mouse *CTP synthetase 1* cDNA sequence was cloned into pLVX-EF1alpha-AcGFP-N1 vector (Clontech, 631983). CTPsyn1-GFP and GFP vector were introduced into cells with jetPEI (polyplus) transfection reagents following manufacturer's protocol.

### Immunofluorescence and microscopy

All cultured cells were fixed in 4% paraformaldehyde in PBS for 10 minutes and washed with PBT (1× PBS + 0.5% horse serum + 0.3% Triton X-100) before imaging. *Drosophila* larval tissues were obtained from age-matched animals raised on apple juice agar plates supplemented with wet yeast. All tissues were dissected into Grace's Insect Medium, fixed in 4% paraformaldehyde in PBS for 10 minutes and washed with PBT. Samples were incubated in primary and secondary antibodies consecutively overnight with intervening washes in PBT. Primary antibodies used were rabbit anti-CTPsyn (Santa Cruz 134457), mouse anti-miranda (gift from Fumio Matsuzaki). Rabbit anti-CTPS antibody (1:500, GeneTex, GTX105265) was used for staining in human cells. Secondary antibodies used were: donkey anti-rabbit Cy5 (Jackson 711-175-152), donkey anti-goat alexa 488 (molecular probes A11055), donkey anti-goat 549 (Jackson 711-585-152). Hoescht 33342 (1 µg/ml) was used to label DNA. All samples were imaged using a Zeiss LSM 510 META confocal microscope. Image processing and analysis was conducted using ImageJ.

### Generation of CTPsyn expression constructs and mutants

A *metallothionein* (*MT*) promoter sequence was amplified from pMT/V5-HisA using primers containing adaptors with BglII and EcoRV digestion sites with Expand high fidelity DNA polymerase. The amplified promoter sequence and the pAVW destination vectors from the *Drosophila* gateway vector collection (obtained from the *Drosophila* Genomics Resource Centre, Bloomington, IN) were digested using BglII and EcoRV (NEB) and ligated together with the amplified pMT promoter to generate the pMT-VW expression vector. *CTPsyn* was cloned into pMT-VW using the Gateway recombination system (Invitrogen). Single base substitutions were made in the pMT-VW-*CTPsyn* expression vector (decribed above) using a quickchange PCR protocol. PCR primers were designed to include nucleotide substitutions of interest. PCR was performed on the pMT-VW-*CTPsyn* expression vector using Phusion high fidelity polymerase (NEB). Following amplification, template DNA was digested using Dpn1. Plasmid DNA was purified using a MinElute low volume PCR purification kit (Qiagen) and transformed into *E. coli DH5α* cells.

### Drosophila genetics

To overexpress *CTPsyn* in various tissues, *w ; pUASp-CTPsyn/Cyo* flies (as described by Azzam and Liu, 2013) were crossed to the *Act5c-Gal4* driver (Bloomington *Drosophila* stock centre) for ubiquitous expression. For neuroblast overexpression *Pros-GAL4* was obtained from the putative-enhancer collection (Bloomington *Drosophila* Stock Centre [BDSC] at Indiana University, USA). Flies expressing *UAS* driven shRNA targeting *AKT1 (P(KK100495)VIE-260B*; Vienna *Drosophila* RNAi Center, Vienna) were crossed to flies with a *GAL4* driver expressed in neuroblasts (*Insu-GAL4*, gift from Jürgen Knoblich) (*AKT1* RNAi line previously characterised by Lee et al., 2013).

### Analysis of *Drosophila* neuroblasts and imaginal tissues

*w^1118^* larval CNS and imaginal discs were dissected for all wild-type experiments. Well-fed larvae were reared on apple juice plates with standard medium comprising of 80 g/l maize, 18 g/l dried yeast, 10 g/l soya flour 80 g/l malt extract, 40 g/l molasses, 8 g/l agar, 6.6 ml acid mix (comprising 50% propionic acid and 3.2% phosphoric acid)/l throughout larval life. For larvae on a restricted diet, larvae were removed at 5 hours and 24 hours for CNS and imaginal disc studies respectively, and placed on apple juice plates. CNS were imaged at 24 hours after larvae were removed from food (L2 stage) and imaginal discs were imaged 52 hours after removal from food (Stage L3). For larval starvation and re-feeding assays 1st instar larvae were starved for 1 day, then transferred to food and the number of neuroblasts containing cytoophidia were scored after 0, 1 and 5 hours. CNS and imaginal tissues were dissected, and fixed for 10 min in 4% paraformaldehyde. Immunofluorescence was performed using rabbit anti-CTP synthase y88 (sc-134457, 1:1000 Santa Cruz BioTech), rabbit anti-Miranda (1:200, gift from C. Doe,), guinea pig anti-Dpn (1:10,000, gift from J. B. Skeath), as previously described (Grice and Liu, 2011).

### Western blotting

Cell lysates were run on a 12% polyacrylamide gel and transferred to a PVDF membrane (GE Healthcare). To prevent non-specific binding of antibody, membrane was incubated in blocking buffer (5% milk in PBS) for 1 hour in room temperature. After blocking, the membrane was incubated in primary antibodies overnight at 4°C. Primary antibodies: rabbit anti-CTPS IgG antibody (GeneTex, GTX105265) and mouse anti-αTubulin IgG (Sigma, T5168) were diluted in blocking buffer in 1:4000 and 1:10,000 dilution, respectively. For *Drosophila* western blots (supplementary material Fig. S3, Fig. S4B) rabbit anti-Histone H3 antibody (abcam ab1791) was used for the loading control (1:10,000). Following 3 washes with 1% Tween-20 in PBS, the membrane was incubated in secondary antibodies at room temperature for 1 hour. Secondary antibodies: HRP-conjugated anti-rabbit IgG (GeneTex, GTX77137) and anti-mouse IgG (PerkinElmer Life Sciences Inc., NEF822), were diluted in blocking buffer in 1:2000 and 1:20,000 dilution, respectively. The bound antibodies were detected with Luminata^TM^ Forte Western HRP Substrate (Millipore, WBLUF0500) and X-ray film. Relative protein abundances were measured using imageJ.

### Quantification of intracellular nucleotides

7×10^6^ cells were washed with 1× phosphate buffer (pH 7.4) and lysed in 80% methanol. After centrifugation at 12,000 r.p.m. for 10 min at 4°C, supernatants were dried and kept at −80°C before injection. The pellet was resuspended in 200 µl water. 5 µl aliquots were injected onto a separation column (ACQUITY UPLC BEH C18, 1.7 µm, 2.1×100 mm reversed-phase column, Waters) with a flow rate of 0.5 ml min^−1^ and analysed with an Acquity Ultra Performance Liquid Chromatography (UPLC Waters) interfaced with PDA photodiode Array (Waters). Mobile phase A was 50 mM TEAA with 4 mM EDTA, pH 7.0 and mobile phase B, 100% acetonitrile. A programmed mobile phase-gradient was used during a 10-min run: 0 min, 0.1% B; 6.8 min, 2% B; 6.9 min, 0.1% B; 10 min, 0.1% B. The concentration of the four nucleotides ATP, UTP, CTP and GTP was quantified at 270 nm absorbance. Concentrations were determined by using calibration curves of the four nucleotides. The linearity gave a correlation coefficient of the linear regression curves greater than 0.99 for the four nucleotides. Peak areas were integrated, and the amounts calculated were as follows: ATP, 4.7 min; UTP, 2.7 min; CTP, 2.1 min, and GTP, 1.5 min.

### Metabolomic profiling

Young adult female flies are fed with wet yeast for 2 days before the collection. The following three groups of flies were collected: 1) Control group – *y,w* flies fed with normal food; 2) Inhibition group – *y,w* flies fed with food containing 1 mg/ml DON to inhibit CTPsyn activity; 3) Overexpression group – globally overexpressing *CTPsyn* using an *actin-GAL4* driver. These flies were subdivided into 30 subgroups with 100 flies in each subgroups. In total 3000 flies were collected for the metabolic profiling. Whole flies were frozen in liquid nitrogen and shipped to Metabolon, Inc (Durham, North Carolina, USA) for metabolomic profiling.

## RESULTS

### Cytoophidium assembly occurs in response to nutrient stress

**Fig. 1.**
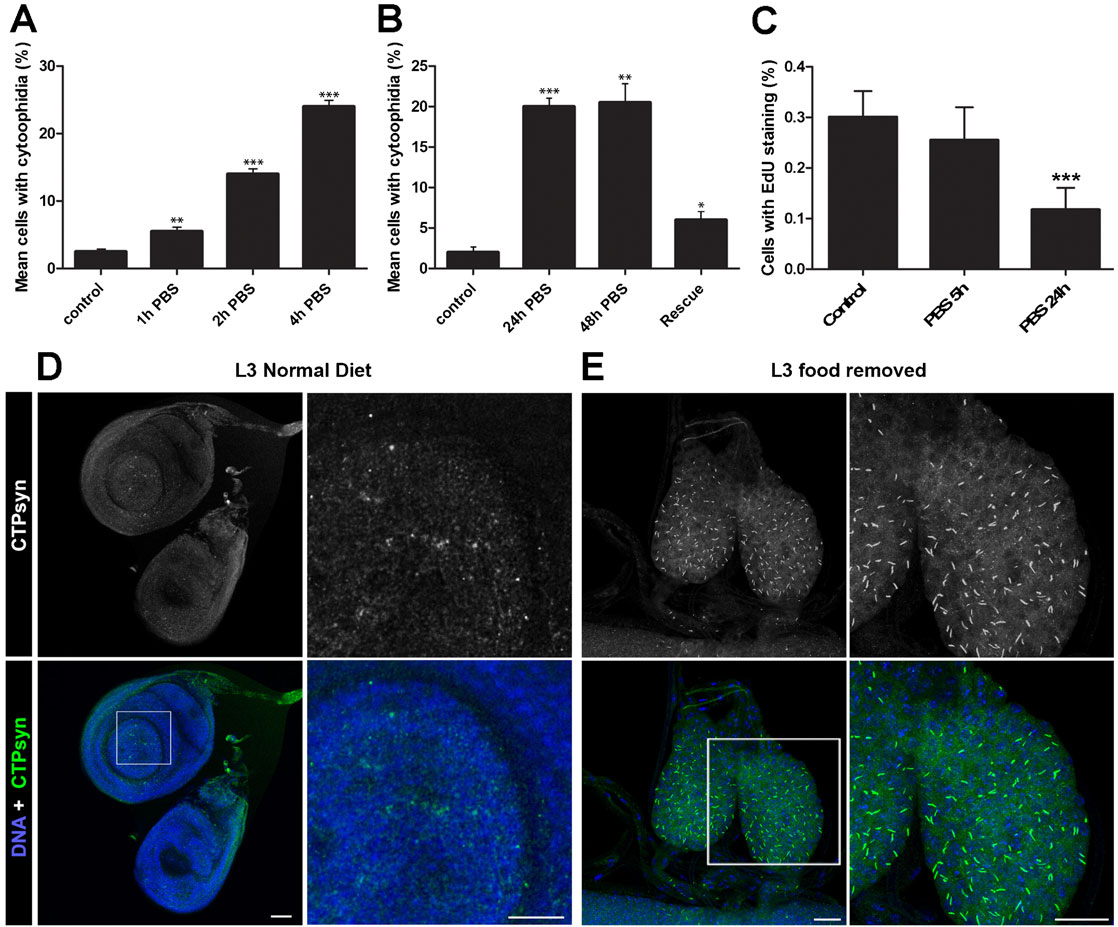
Cytoophidium assembly occurs in response to nutrient stress. (A) Mean number of cells containing visible cytoophidia increases in nutrient restricted cells. Significantly more cytoophidia are seen in S2R+ cells in PBS after just one hour. Control cells are maintained in complete *Drosophila* Schneider's medium. At least 100 cells were counted from ten sites per well from at least three independent replicates. (B) Cells with increased numbers of cytoophidia following nutrient restriction can be rescued by addition of whole media. Cells were examined two hours after re-introduction of whole media. (C) No significant change in EdU marked cells is observed after 5 hours, indicating that cell cycle arrest is not necessary for cytoophidia formation in S2 cells. (D) Response to nutrient restriction in imaginal disc tissue *in vivo*. Imaginal discs of L3 larvae display only diffuse cytoophidia when fed a normal diet. (E) In larvae raised on a nutrient restricted diet, the imaginal discs are smaller and cytoophidia are highly prevalent. Scale bars: 10 µm. ***P<0.001, **P<0.01, *P<0.05.

It was previously shown that *S. cerevisiae* cells grown in media lacking glucose exhibited greater numbers of CTPsyn filaments (Noree et al., 2010). This effect could be reversed by addition of glucose. To see if the response to nutrient stress is evolutionarily conserved, we removed cell culture medium from *Drosophila* S2R+ cells in logarithmic growth and incubated with PBS. Cells were examined by immunofluorescence at various time points to determine the effect of nutrient stress on cytoophidium assembly. A significant increase in number of cytoophidia was seen after one hour, the shortest interval measured. Numbers of cells with visible cytoophidium formation reached a maximum of around 20%, after 4 hours in PBS (Fig. 1A). The observed response of cytoophidia to nutrient stress could be reversed by the re-addition of whole media (Fig. 1B). It remained unclear whether the increased cytoophidium formation displayed by nutrient restricted cells was a consequence of cell cycle arrest. We performed Edu staining to label S-phase cells in nutrient restricted cells to give an indication of the number of cells exiting the cell cycle. No significant change in Edu staining was seen following 5 hours of nutrient restriction, indicating that cell cycle arrest is not necessary for cytoophidium formation in cells undergoing nutrient restriction (Fig. 1C).

To investigate whether nutrient induced change in CTPsyn localisation was conserved in animal tissues *in vivo*, we raised larvae on food for 24 hours, transferred them onto minimal food for 52 hours, and then dissected. The time analysed corresponded to the third instar stage. Imaginal discs, including wing, leg, and haltere, display diffuse CTPsyn staining and exhibited no visible cytoophidia in well fed larvae (Fig. 1D). In nutrient restricted larvae, cytoophidia were abundant in the imaginal discs (eye, wing, leg and haltere) of third instar larvae, which were also reduced in size (Fig. 1D, showing imaginal discs from a nutrient restricted larva). In summary, CTPsyn localisation changes as part of an adaptive metabolic response to inactivate cytoplasmic CTPsyn during nutritional stress *in vivo*.

### Redistribution of CTPsyn occurs during *Drosophila* neurogenesis

**Fig. 2.**
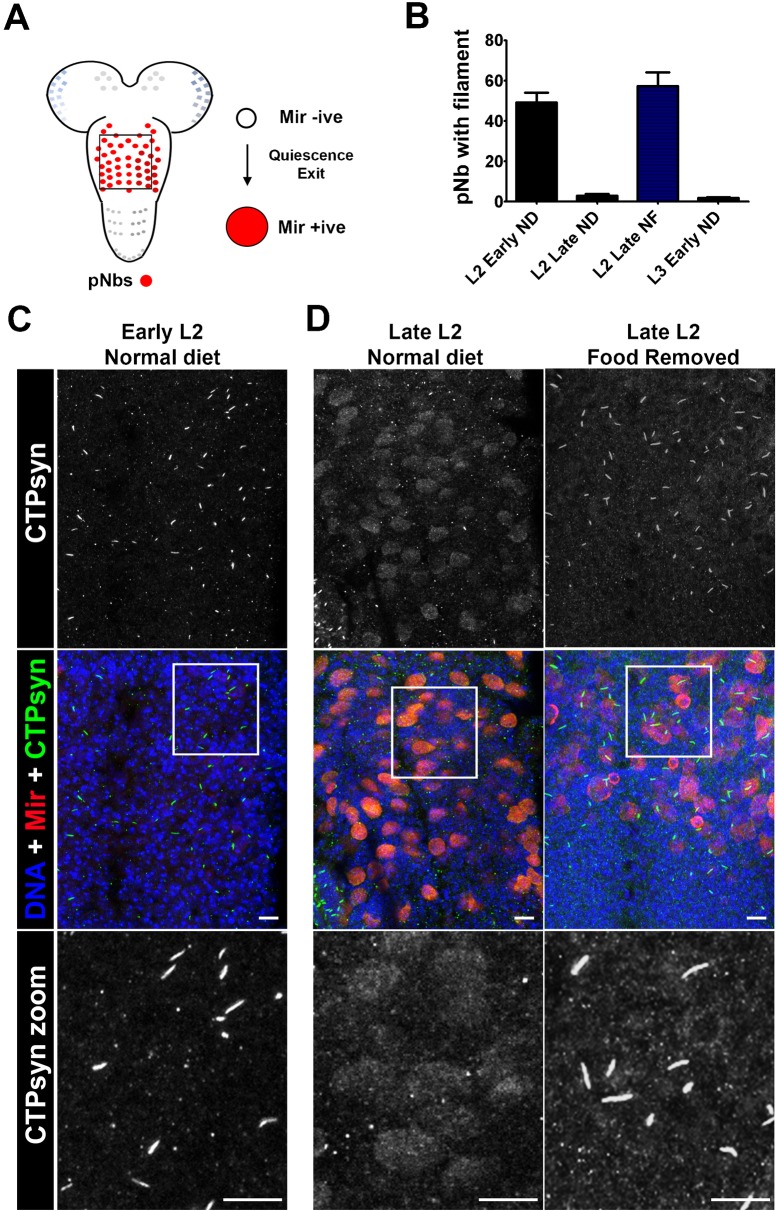
CTPsyn distribution in *Drosophila* larval post embryonic neuroblasts (pNbs). (A) A schematic of the larval CNS showing the location of the pNbs. The box represents the section of the CNS represented in panels C and D. The majority of larval pNbs exit quiescence during the late L1 (1^st^ instar) and early L2 (2^nd^ instar) stages. This is characterised by pNb enlargement and the expression of markers such as Miranda (Mir). (B) Counts of pNbs containing cytoophidia in L2 early, L2 late and L3 larvae early reared under different feeding conditions (ND, normal diet; FR, food removed). n>10 animals per group. (C) CTPsyn localisation in early and late L2 when on a normal diet (see Materials and Methods). CTPsyn forms cytoophidia in quiescent pNbs (Mir negative). (D) As neuroblasts exit quiescence (late L2, normal diet) and begin to divide CTPsyn becomes diffuse. (E) CTPsyn localisation in late L2 when food is removed (late L2, food removed) after 24 hrs. CTPsyn aggregates into filaments when nutritional stresses are present. These images are representative of all 10 animals imaged. Scale bars: 10 µm.

Having seen that cytoophidium formation was responsive to nutrient stress *in vivo* we asked whether cytoophidium formation may be required during normal developmental processes. The post embryonic neuroblasts of the *Drosophila* CNS have previously been shown to exhibit high levels of cytoplasmic (i.e. non-filamentous) CTPsyn (Chen et al., 2011). The majority of these neuroblasts remain in a quiescent state during the early 1^st^ instar larval stage (Fig. 2A). When the larvae have the required access to food, neuroblasts exit quiescence and re-enter the cell cycle during late 1^st^ (L1) and early 2^nd^ instar (L2) stages (Chell and Brand, 2010; Sousa-Nunes et al., 2011; Truman and Bate, 1988). As the demand for nucleotides is upregulated in proliferating cells, we hypothesised that there may be a concomitant switch in cytoophidium assembly/disassembly. We dissected 1^st^ and 2^nd^ instar larvae and investigated CTPsyn distribution by immunofluorescence. In well fed larvae, CTPsyn can be observed in filaments in the early 3^rd^ instar thoracic ganglion, however these filaments disappear as larvae develop through L2 and L3 stages (Fig. 2A,B). The cytoophidia appear on the ventral surface in cells corresponding to the regions where the quiescent neuroblasts reside (Fig. 2C). As neuroblasts exit quiescence they enlarge and begin to divide. Following this process CTPsyn filaments disappear, and CTPsyn is localised in the cytoplasm in a diffuse pattern (Fig. 2D).

### Nutritional restriction during neurogenesis leads to cytoophidium formation

**Fig. 3.**
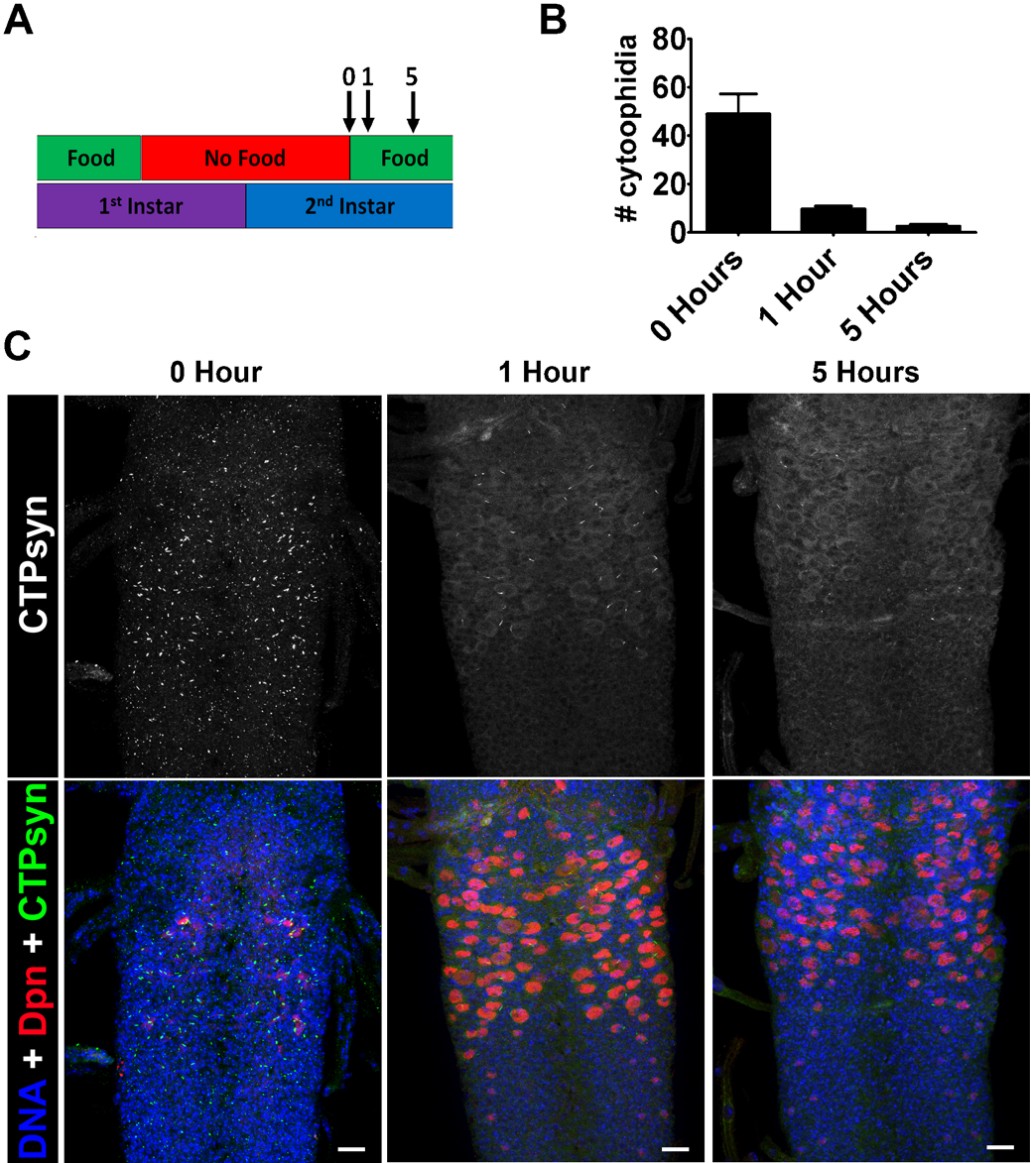
Re-feeding starved larvae promotes cytoophidia disassembly. (A) 1^st^ instar larvae were starved for 1 day. Larvae were then transferred to food and the number of neuroblasts with cytoophidia were scored after 1 and 5 hours. (B) Re-feeding induces the dissociation of the cytoophidia in neuroblasts that accumulated during starvation. After 5 hours cytoophidia numbers are comparable with those seen in well fed L2 larvae. (C) Representative pictures CNS from larvae that have been re-fed for 0 hr, 1 hr and 5 hrs. These images are representative of >6 animals imaged. Scale bars: 10 µm.

Neuroblasts continue to divide throughout larval life and CTPsyn remains diffuse in the neuroblast cytoplasm throughout this period (Chen et al., 2011). Nutritional stress during this period has been shown to affect neuroblast division and cell cycle entry. For example, when young larvae are starved before a critical weight period is reached (during the L3 stage), developmental progression and neurogenesis is halted (Tennessen and Thummel, 2011). To model nutritional stress, and to stall growth and neurogenesis, we transferred newly hatched larvae from basic food to apple juice plates, after 12 hours. We then reared the larvae on apple juice plates for 24 hours plates before dissecting. In these starved larvae, cytoophidia can be found in the majority of neuroblasts (Fig. 2E). We next looked to see how the re-addition of food, and thus the re-initiation of neurogenesis, affects the presence of cytoophidia (Fig. 3). 1st instar larvae were starved for 1 day, and then transferred to food. The number of neuroblasts containing cytoophidia was scored after 0, 1 and 5 hours (Fig. 3A). We found that re-feeding induced the dissociation of the cytoophidia in neuroblasts, which had accumulated during the starvation period (Fig. 3B,C). In addition, 5 hours after the re-addition of the normal diet, cytoophidia numbers were comparable with those seen in well fed L2 larvae. Furthermore, strong cytoplasmic staining was seen to reappear in the neuroblasts of these animals (Fig. 3C). These data again suggest that CTPsyn localisation changes as part of an adaptive metabolic response to inactivate cytoplasmic CTPsyn during nutritional stress in neuroblasts.

**Fig. 4.**
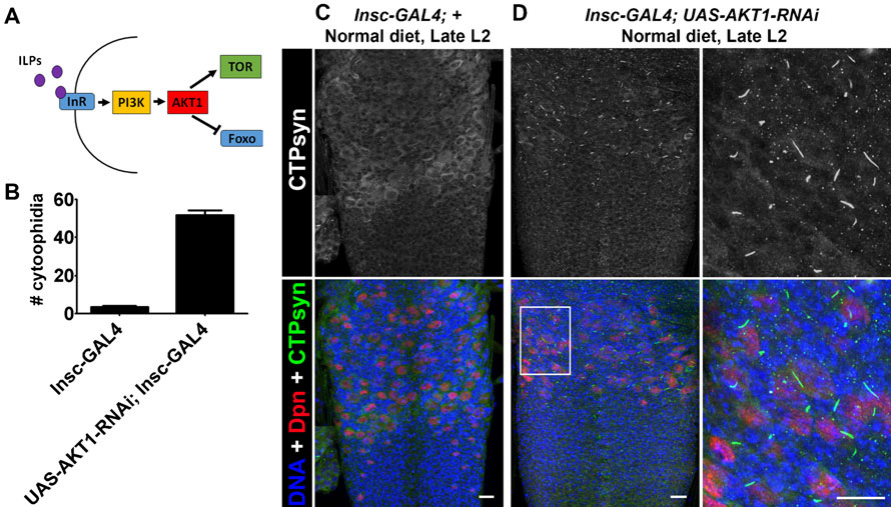
*AKT1* knockdown induces Cytoophida formation. (A) When nutrients are available insulin-like peptides (ILPs) bind the insulin receptor (InR), activating the PI3K/AKT pathway. This in turn inhibits the growth inhibitor Foxo and activates TOR, leading to growth and division of cells, including neuroblasts. (B) *AKT1* was knocked down in neuroblasts using *Insc-GAL4, UAS-AKT1-RNAi* to mimic nutritional stress. *AKT1* knockdown induces cytophidia formation. (C) *Insc-GAL4* controls displayed very few neuroblasts with cytophidia. (D) Cytophidia in *Insc-GAL4, UAS-AKT1-RNAi* neuroblasts. These images are representative of >6 animals imaged. Scale bars: 10 µm.

To mimic nutritional stress, we also knocked-down the serine-threonine kinase *AKT1* in neuroblasts using *Insc-GAL4, UAS-AKT1-RNAi*. During L1 and L2, normal food intake activates insulin signalling and the PI3K/AKT pathway. This in turn activates TOR, and represses the growth inhibitor Foxo, leading to the initiation of neuroblast division and cell proliferation (Fig. 4A) (Chell and Brand, 2010; Goberdhan and Wilson, 2003; Sousa-Nunes et al., 2011). *Insc-GAL4, UAS-AKT1-RNAi* neuroblasts have increased numbers of cytoophidia when compared to controls (Fig. 4B–D), suggesting that inactivation of the AKT1 pathway induces cytoophidia formation. The prolonged presence of cytoophidia in the neuroblast however, appears to have no detrimental effect. Overexpression of *CTPsyn* in the neuroblasts (*Pros-GAL4, UAS-CTPsyn-A*) generates extremely large cytoophidia, but this has no overt effect on overall development (supplementary material Fig. S1). In summary, cytoophidia are formed in neuroblasts that are quiescent, or in neuroblasts from the CNS of starved and developmentally stalled larvae. These results are consistent with the hypothesis that CTPsyn in cytoophidia is inactive.

### Either inhibition or overexpression of *CTPsyn* promotes cytoophidium assembly

We have previously shown that in human cells that the proportion of cells containing visible cytoophidia is low during logarithmic growth under standard conditions, with around 5% of cells displaying filaments (Chen et al., 2011). Filaments could be induced to form when cells were treated with the glutamine analogues 6-Diazo-5-oxo-L-norleucine (DON) and azaserine, which both act as competitive inhibitors of CTPsyn.

In order to investigate whether *Drosophila* cells displayed similar properties to human cell lines, S2R+ cells were treated with varying concentrations of DON. In untreated cells very low endogenous numbers of cytoophidia (≈3%) could be detected by immunofluorescence, whilst significant increases were observed in DON-treated cells as previously reported (supplementary material Fig. S2A–C). Furthermore, the response of S2R+ cells to DON was proportional to the concentration of drug applied as observed in human cells *in vitro*.

We have reported that only one isoform of *Drosophila* CTPsyn is able to form cytoophidia (Azzam and Liu, 2013). Ectopically expressing the cytoophidium-forming isoform of CTPsyn can induce cytoophidia in many tissues in *Drosophila* (Azzam and Liu, 2013). To see whether expression of *CTPsyn* would also induce cytoophidium formation in cell culture, *CTPsyn* was cloned into an expression vector under the control of a *metallothionein* promoter with a venus (EYFP) fluorescent tag (referred to as CTPsyn-YFP hereafter). This expression construct was transfected into *Drosophila* S2R+ cells and stable lines established in order to further investigate the formation of cytoophidia in vivo.

To determine whether expression of the *CTPsyn-YFP* transgene had an adverse effect on cell survival, cells were assayed for viability using a trypan blue dye exclusion assay. Loss of cell viability has been reported at concentrations exceeding 1 mM (Southon et al., 2004). Transgene expression was induced using 1 mM CuSO_4_ to achieve robust expression without affecting cell viability. No discernible difference in viability was observed in cells in which transgene expression was induced by CuSO_4_ for 8 hours, compared to uninduced or wild-type S2R+ cells indicating that *CTPsyn* overexpression has no adverse effect on the health of cell cultures (supplementary material Fig. S2E). Cytoophidium forming properties of the developed transgenic cell line were examined over time after CuSO_4_ induction, by fixing cells in paraformaldehyde and imaging at various timepoints. Cytoophidia were visible at very low levels after 4 hours post induction in less than 5% of cells. Cytoophidia were visible in 100% of cells after ten hours of incubation with CuSO_4_ (supplementary material Fig. S2D). The mean cytoophidia area increased with time. The average number of filaments per cell increased very rapidly between four and five hours, then decreased until eight hours, during which time the mean filament size increased significantly due to fusion of smaller cytoophidia into larger structures as previously reported (Gou et al., 2014) (supplementary material Fig. S2F). Together, these data indicate that cytoophidium formation is proportional to CTPsyn concentration.

### Analysis of *CTPsyn* overexpression on metabolic profiles

In previous studies, we have shown that overexpression of *CTPsyn* or treatment with small molecule inhibitors is sufficient to induce abundant cytoophidia formation in *Drosophila* tissues *in vivo* (Azzam and Liu, 2013; Chen et al., 2011). In order to investigate the effect of altered CTPsyn concentrations and cytoophidia abundance on cellular physiology, we conducted metabolomic profiling on flies either overexpressing *CTPsyn*, treated with DON, or wild-type controls. We obtained global metabolome profiles covering 308 metabolites in three groups of *Drosophila*: flies with *CTPsyn* overexpression (*Oe*, as described by Azzam and Liu, 2013), wild-type flies treated with CTPsyn inhibitor DON (*In*), and wild-type flies with no treatment as a control (*Wt*).

**Fig. 5.**
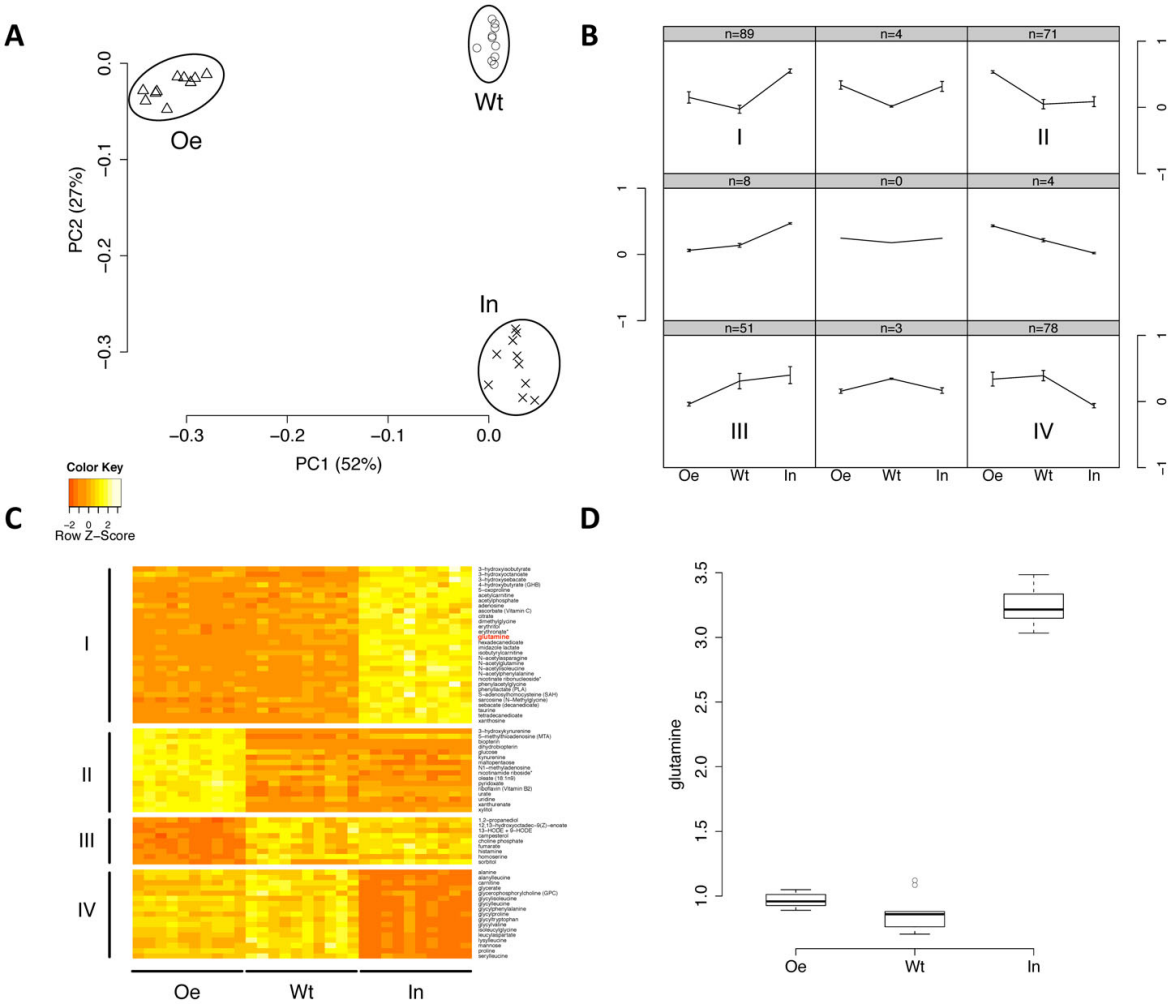
Global metabolomic profiling indicates negligible changes in CTPsyn activity *in vivo*. (A) Principle component analysis shows that the samples of wild-type (Wt), CTPsyn inhibition (In), and *CTPsyn* overexpression (Oe) groups are clearly segregated. Two principal components (PC1 and PC2) are plotted and their proportions of variance are labeled. (B) The mean values of compounds in each state were normalized to (−1,1) and clustered into nine patterns by self-organizing mapping. The numbers of compounds in each pattern are showed on the top of the grids. The patterns that contain the most compounds are indicated by (I, II, III, V). (C) The heat map shows metabolites in major patterns that have significantly differential levels in three groups. Seventy-two metabolites in major patterns of (B) SOM (I, II, III, IV) were selected by one-way ANOVA with Bonferroni adjusted P<10^−5^. (D) The levels of glutamine in three groups are shown in the boxplot. Glutamine belongs to pattern I and is also colored in red in panel C.

Principle component analysis of metabolome data showed that the three groups are clearly distinct from each other in terms of their global metabolite levels (Fig. 5A). We then used a self-organizing map (SOM) to reveal the relative metabolite changes among the three groups for all metabolites. As shown in Fig. 5B, four major patterns emerge: low–low–high (I, upper left), high–low–low (II, upper right), low–high–high (III, lower left), and high–high–low (IV, lower right) in *Oe*, *Wt*, and *In* group respectively. We found that the metabolites involved in amino acid metabolism are enriched in pattern I (P = 0.005, Fisher's exact test), lysolipids are enriched in pattern II (P = 0.0008) and dipeptides are enriched in pattern IV (P = 0.0001).

To ensure that these metabolites show statistically significant differences among the three groups, we applied one-way ANOVA to further select the metabolites (Bonferroni adjusted P<10^−5^) belonging to the four major patterns (Fig. 5C). For the metabolites in patterns I and IV, the treatment of CTPsyn inhibition strongly influenced their levels compared to the control while the mutants with *CTPsyn* overexpression have no difference compared to the control. Glutamine belongs to pattern I and shows increased level in *In* compared to *Wt* and *Oe* (Fig. 5D). This can be explained by the fact that the inhibition of glutaminase activity of CTPsyn may lead to the accumulation of glutamine. The similar levels of glutamine between *Oe* and *Wt* groups suggest that the glutaminase activity of CTPsyn remains the same even after the overexpression of *CTPsyn*. These two major patterns support the idea that the CTPsyn sequestered in the cytoophidia filament has no CTPsyn-related enzymatic activity. For the metabolites in patterns II and III, the overexpression of *CTPsyn* strongly influenced their levels compared to the control and the group with CTPsyn inhibition. These groups of metabolites are unrelated to the canonical enzyme function of CTPsyn as they show no difference with the control after the inhibition of CTPsyn. These changes may be accounted for by the presence of Gal4 in the *Oe* group. However, some changes may be the result of metabolic functions of cytoophidia. In conclusion, our analysis of metabolome data supports the hypothesis that the formation of cytoophidia could sequester CTPsyn enzyme activity.

### Overexpression of *CTPsyn* only moderately increases CTP pool in human cells

**Fig. 6.**
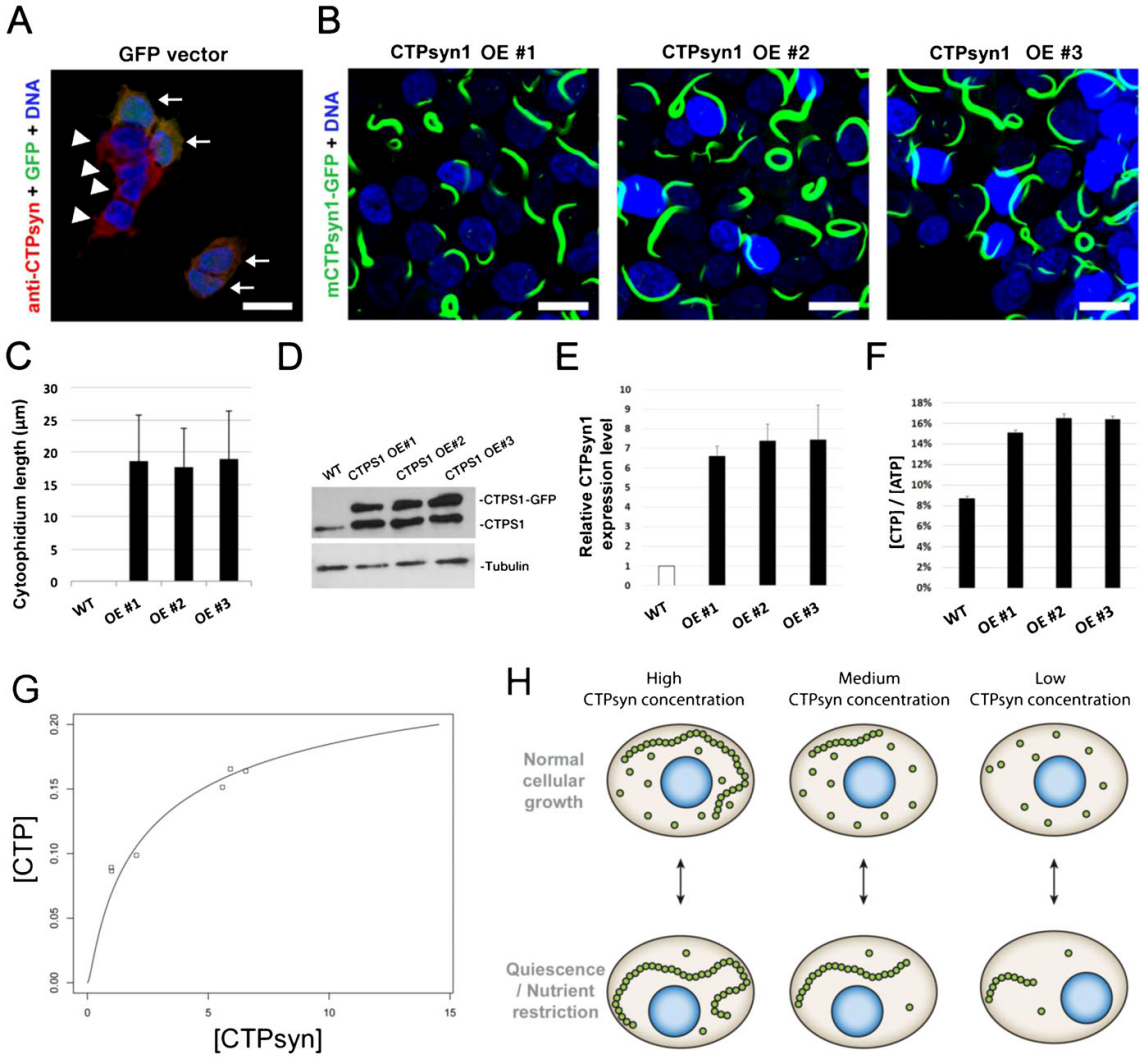
CTPsyn, cytoophidia and CTP production. (A) Cytoophidia are undetectable in wild-type and GFP vector transfected human 293T cells. (B) The mCTPsyn1-GFP proteins were stably expressed by CTPsyn1 OE#1, CTPsyn1 OE#2 and CTPsyn1 OE#3 cell lines. Almost every cell contains one or more cytoophidia. (C) Quantification of length of cytoophidia expressed by three independent cell lines. (D) Western blot of CTPsyn1 protein in wild-type, and mCTPsyn1-GFP expressing 293T cell lines. (E) Quantification of the relative protein abundance of endogenous CTPsyn1 and exogenous mCTPsyn1-GFP in CTPsyn1 OE#1, CTPsyn1 OE#2 and CTPsyn1 OE#3 cell lines. (F) The relative CTP concentration in cell lysates of wild-type, and three *CTPsyn1* overexpression 293T cell lines. Overexpression of *CTPsyn* moderately increased intracellular CTP concentration in human cells. (G) The fitting of total CTPsyn and CTP concentration using the non-linear relationship derived from our mathematical model (see text). The x axis is total CTPsyn and y axis is CTP concentration. Data points represent experimentally measured values in wild type and *CTPsyn* overexpressing human 293T cells. (H) Proposed model of cytoophidia assembly in response to nutrient or developmental conditions. Scale bars: 20 µm.

According to previous studies, manipulations of certain conditions, such as glutamine deprivation or inhibition of GTP synthesis with drugs, could stimulate cytoophidium formation in some cells (Calise et al., 2014; Gou et al., 2014). To investigate the function of the cytoophidium in mammalian cells, 293T cells were transfected with a construct of mouse CTP synthase 1-GFP fusion protein (mCTPsyn1-GFP) which is driven by the *EF1α* promoter. Expression of mCTPS1 promoted assembly of cytoophidia in 293T cells in normal culture condition (Fig. 6A,B). Cytoophidia in mCTPS1-GFP expressing cells were significantly longer than those in normal 293T cells with or without DON treatment (Fig. 6B,C; supplementary material Fig. S4), indicating that more CTPsyn proteins accumulated in cytoophidium structure in cells expressing *mCTPsyn1*. We generated three stable cell lines overexpressing mCTPsyn1-GFP at approximately 6–8-fold higher than endogenous levels. (Fig. 6D,E). However, the intracellular CTP concentration only increased to 2-fold higher than that of wild-type cells indicating that CTPsyn activity does not increase linearly with protein concentration (Fig. 6F).

### Mutations at the CTPsyn oligomer interfaces alter cytoophidia assembly

End product inhibition of CTPsyn by CTP is critical for the regulation of its enzymatic activity (Aronow and Ullman, 1987; Endrizzi et al., 2005; Long and Pardee, 1967). Previous experiments have shown that a mutation at the CTP binding site of CTPsyn in *S. cerevisiae* inhibited end product inhibition by CTP (Ostrander et al., 1998). Site-directed mutagenesis was used to construct a point mutation at the equivalent conserved *CTPsyn* residue (*CTPsyn*^E160K^) in CTPsyn-YFP expression vector (supplementary material Fig. S2). When *CTPsyn*^E160K^ was transfected into *Drosophila* S2R+ cells, cytoophidium formation was seen to be completely disrupted, although diffuse cytoplasmic YFP could be seen, along with numerous punctate structures (supplementary material Fig. S5). Similar results have previously been reported in *S. cerevisiae* indicating that regulation of cytoophidium formation by end-product inhibition is an evolutionarily conserved phenomenon (Noree et al., 2010).

Allosteric binding of CTP has been shown previously to induce CTPsyn tetramer formation (Long and Pardee, 1967). It has also been demonstrated that treatment with the CTPsyn inhibitor, 6-Diazo-5-oxo-L-norleucine (DON), reduced the amount of CTPsyn tetramerisation in *E. coli*, which suggests that the tetramer form of the enzyme may be excluded from cytoophidia (Robertson, 1995). From these data it is unclear whether CTPsyn monomers, dimers or tetramers are incorporated into cytoophidia. We therefore decided to investigate the effect of mutations influencing residues at the tetramer or dimer interface of CTPsyn on cytoophidium assembly (supplementary material Fig. S4). We constructed point mutations in CTPsyn-YFP at the tetramer interface (*CTPsyn*^G151E^ and *CTPsyn*^R163H^) and assayed for filament formation in S2R+ cells. Both *CTPsyn*^G151E^ and *CTPsyn*^R163H^ resulted in significantly longer cytoophidia when expression was induced in S2R+ cells compared to wild type protein controls (supplementary material Fig. S5). These data indicate that tetramer formation may not be necessary for CTPsyn to form cytoophidia. To determine whether CTPsyn dimer formation is necessary for cytoophidium formation we constructed point mutations at the monomer–monomer interface, predicted to disrupt dimer formation (*CTPsyn*^V114F^ and *CTPsyn*^M156I^). Both *CTPsyn*^V114F^ and *CTPsyn*^M156I^ resulted in significantly shorter cytoophidia when expressed in S2R+ cells (supplementary material Fig. S5). Cytoophidium formation was not disrupted completely as in *CTPsyn*^E160K^. It is unclear to us whether this is due to incomplete disruption of dimer formation in *CTPsyn*^V114F^ and *CTPsyn*^M156I^. To determine whether changes in cytoophidia formation were due to changes in CTPsyn abundance, we performed western blots to detect the relative amounts of expressed proteins for each mutant. Comparison of relative CTPsyn abundances indicates that most of the mutant proteins did not result in an apparent change in protein abundance. However, CTPsyn^R163H^ and CTPsyn^E160K^ appeared to have significantly lower protein levels. CTPsyn^R163H^ displayed longer cytoophidia than wild type, despite lower protein abundance, whereas CTPsyn^E160K^ displayed significantly less cytoophidia and had clear morphological differences. Therefore, we conclude that CTPsyn increase is not a significant confounding factor in the interpretation of these phenotypes. Together these results imply that CTPsyn dimers may be the multimer subunits of cytoophidia, whilst tetramers may not necessarily be required for cytoophidium assembly.

### A simple mathematical model of cytophidium formation

Two types of cytoophidia have been described in *Drosophila* germline cells, distinguished by their relative sizes; micro-cytoophidia and macro-cytoophidia (Liu, 2010). More recently, we have demonstrated that micro-cytoophidia can undergo multiple rounds of fusion to form macro-cytoophidia in mammalian cells (Gou et al., 2014). Here we developed a simple mathematical model to describe the incorporation of CTPsyn into cytoophidia. First, we only consider the micro-cytoophidia of size 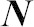 polymerised sequentially at both ends from CTPsyn n-mers (n = 2 for dimer and n = 4 for tetramer). The kinetic constants of nucleation and de-nucleation of n-mers in the filament are 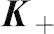 and 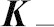 respectively. Under biochemical equilibrium, the numbers of filaments of length 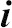, 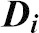, satisfy:
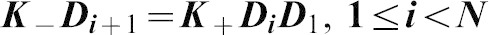


This leads to the exponential distribution of 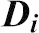: 
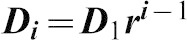
, where 
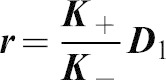
.

Here n-mers are formed from CTPsyn monomers with kinetic constants: 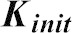 and 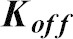 respectively. Therefore 
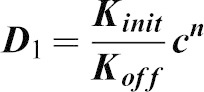
 where 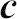 is the number of CTPsyn monomers. The total amount of CTPsyn is the sum of free CTPsyn monomers and those sequestered in filaments:
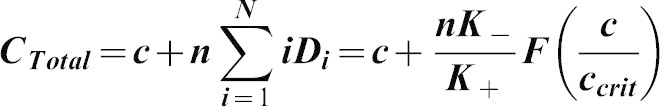


Here 
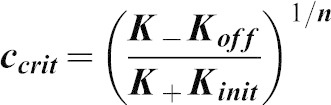
is the critical number of free CTPsyn monomers and 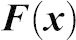 is
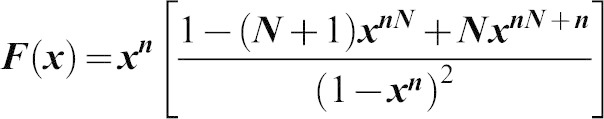


From the above equation, we can derive the relationship between free CTPsyn monomers and total amount of CTPsyn. For a cytoophidium of size 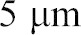 and CTPsyn protein of size 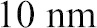, we estimated 
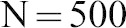
. When 
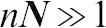
, 
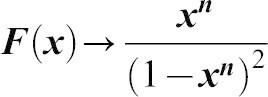
 for 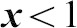. When 
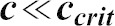
, 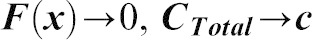 and all CTPsyn are in the form of monomers. When 
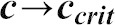
, 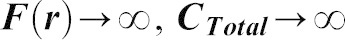. In other words, ***c*** is kept close to 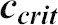 when 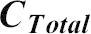 is significantly overexpressed.

Our model can be readily generalised to include the CTPsyn in toroidal cytoophidia by considering the circularisation of linear cytoophidia as additional reversible biochemical reactions. Because circular cytoophidia are in equilibrium with linear cytoophidia of the same length and will not further increase in sizes, the inclusion of circular cytoophidia only leads to multiplying the amount of CTPsyn sequestered in filament by a constant. Furthermore, including the formation of macro-cytoophidia from the nucleation of micro-cytoophidia into our model does not change the nature of our result either. It is worth noting that cytoophidia only becomes visible after it is longer than a certain size. In our model the number of filaments exponentially decreases as the size increases. Therefore, the visible filament longer than the detection limit in a single cell can be very small. This explains 1–3 fibres per cells shown by our images while the majority of CTPsyn is sequestered into filaments below the limit of detection.

In order to determine whether our model was applicable to experimental data, we have fitted our model using the measured concentrations of CTP in wild type and *CTPsyn* overexpressing human 293T cells. We assumed that the CTP concentration is proportional to the concentration of catalytic active form of CTPsyn, tetramer 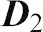, and the cytophidium is formed by polymerization of dimers. According to our model, total CTPsyn and CTP concentration follow the relationship: 
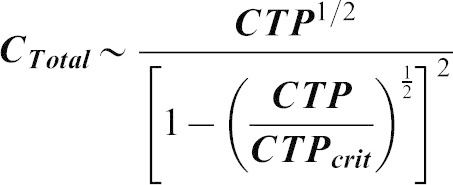
 where 
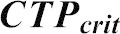
 is the critical value of CTP. We fitted the data by non-linear regression (R^2^ = 0.96) (Fig. 6G).

Our model predicts that CTP concentration does not increase linearly with total CTPsyn and reaches a plateau quickly when CTPsyn is overexpressed. This predicted upper limit of CTP (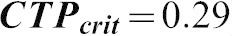) is only 3-fold higher than the measured CTP concentration (0.09) in wild type cells. This indicates that the level of CTP in the cells has been significantly buffered during *CTPsyn* overexpression due to cytophidium formation. This is because most CTPsyn enters into cytoophidia when *CTPsyn* is overexpressed. The amount of free CTPsyn monomers, dimers and tetramers are kept fairly constant but can be quickly released from the filament when CTPsyn is significantly depleted. This suggests that cytoophidia acts as a reservoir to tightly regulate the free active CTPsyn level to mediate cellular nucleotide homeostasis.

## DISCUSSION

We demonstrate that cytoophidia assemble during periods of cellular quiescence or low nucleotide demand as a mechanism to inhibit CTPsyn activity in cultured cells and *in vivo*. Our metabolomic data show that overexpressing *CTPsyn* has no overt effect on metabolic profiles, supporting the hypothesis that the formation of cytoophidia acts to sequester CTPsyn enzyme activity. Therefore cytoophidium formation is a physiologically relevant process during development and in mediating adaptive metabolic responses (Fig. 6H).

### Polymerisation as a strategy for regulation of enzymatic activity

As the rate limiting enzyme in *de novo* CTP biosynthesis, CTPsyn occupies a central regulatory role in nucleotide metabolism. It is therefore unsurprising that it is subject to several complementary regulatory strategies. Not only is CTPsyn regulated by multimer assembly, end product inhibition and other allosteric regulators as previously discussed, it is also subject to phosphorylation by multiple kinases (Chang et al., 2007; Choi and Carman, 2007; Kassel et al., 2010). The discovery that CTPsyn activity is also regulated by assembly into higher-order cytoplasmic filaments adds a further level of sophistication to this already finely controlled metabolic pathway. It is unclear from the data presented here what the purpose of this extra level of enzymatic control is required for, however it is possible to speculate that cytoophidium assembly allows for CTPsyn activity to be rapidly up-regulated when required. The large surface area of a filamentous aggregate such as cytoophidia would allow for easy access by diffusible allosteric regulators in the cytoplasm, thereby facilitating rapid disassembly and upregulation. Punctate structures or vesicle bound enzymes may be subject to less rapid activation by similar mechanisms as the cytoplasmic surface area would be smaller.

Our mathematical model, and supporting data, indicate that cellular CTP pools may increase to a maximum of around 3-fold, regardless of the concentration of CTPsyn. We were unable to detect any overt cellular phenotypes arising from CTPsyn overexpression either in cell culture or *in vivo* models. Therefore, we hypothesise that up to a 3-fold increase in CTP levels has a negligible effect on normal cellular growth and development. This may be considered surprising, given the propensity for tumours to overexpress *CTPsyn*, and the efficacy of CTPsyn inhibitors as anti-neoplastic drugs. Further study will be required to elucidate whether the 3-fold CTP limit is overcome during tumourigenesis or whether these small changes are sufficient to impact cell growth in certain pathological states.

We have focused on the assembly of CTPsyn into cytoophidia in this study, however, it remains unclear the extent to which the novel mechanisms for enzymatic regulation suggested here apply to other recently identified filament forming enzymes. Screening of yeast GFP strain collections have identified at least four novel enzyme filaments which appear to be morphologically similar to cytoophidia, although their functions remain elusive (Noree et al., 2010). Moreover, it is unclear whether all filament forming enzymes are subject to enzymatic downregulation when assembled into cytoplasmic filaments. For example, it has long been known that polymerisation of acetyl Co-A carboxylase (ACC) upregulates enzymes activity, although it is unclear whether ACC forms equivalent macro-scale cytoplasmic structures (Beaty and Lane, 1983a; Beaty and Lane, 1983b; Kleinschmidt et al., 1969; Olivares et al., 2011). Recently, it was demonstrated that glutamine synthase is regulated in a similar manner, indicating that downregulation of activity through polymerisation may be a widespread mechanism by which enzymes are regulated (Petrovska et al., 2014).

### Role of cytoophidium formation in neurodevelopment

It was previously shown that CTPsyn is highly expressed in third instar larval neuroblasts and exhibits a diffuse cytoplasmic localisation (Chen et al., 2011). Here we have shown that cytoophidia are highly prevalent in the quiescent neuroblasts of the early larval CNS. Having shown that CTPsyn activity is likely to be inhibited by incorporation into cytoophidia, we can now speculate that the disassembly of cytoophidia is necessary to synthesis critical nucleotides necessary for the transition of these cells from quiescence to proliferation after re-entering the cell cycle. It is not clear from these data however, whether the dispersal of CTPsyn is regulated by cell cycle regulators acting on CTPsyn itself (e.g. by phosphorylation) or through changes in concentrations of allosteric regulators previously shown to effect cytoophidium formation.

Highly regulated nucleotide metabolism is vital for the development of most metazoan tissues due to the essential requirement for *de novo* nucleotides for DNA synthesis. However, several inborn errors of nucleotide metabolism result in conditions with severe neurological symptoms whilst other tissues are seemingly less affected, such as hypoxanthine–guanine phosphoribosyl transferase (HGPRT) deficiency (Lesch–Nyhan disease) (Lesch and Nyhan, 1964). Therefore understanding the control of nucleotide metabolism in the developing CNS is of particular interest for understanding the mechanisms of hereditary pyrimidine enzymopathies.

The data presented here suggest that as well as being involved in the co-ordination of developmental processes, the compartmentalisation of CTPsyn into cytoophidia is involved in mediating adaptive metabolic responses to nutrient availability in tissue culture and *in vivo*. A protein localisation screen of yeast GFP strains previously identified an unprecedented number of proteins exhibiting cytoplasmic redistribution into punctae during nutrient restriction (Narayanaswamy et al., 2009). This indicates that CTPsyn is not the only protein to be regulated by spatial re-organisation in response to extrinsic signals. Further research will be required to determine whether cytoophidium dissociation is required for cells to re-enter the cell cycle following nutrient stress.

### Conclusion

Although it has been demonstrated previously that CTPsyn activity seems to be intimately linked to its polymerisation state, it has been unclear whether cytoophidia contains catalytically active or inactive CTPsyn. Our study suggests that CTPsyn dimers may be the multimer subunits of cytoophidia, the incorporation of which acts to downregulate CTPsyn enzymatic activity. It is possible that the point mutations studied here, predicted to lie at the interfaces between the CTPsyn monomers have unintended effects on CTPsyn structure or monomer interactions, therefore further structural studies will be required in the future to confirm the presence of CTPsyn dimers within cytoophidia. However, our data are supported by previous observations that treatment with the CTPsyn inhibitor DON causes a reduction in tetramer formation in *E. coli* (Robertson, 1995). As DON results in drastically increased cytoophidia formation, it follows that tetramers are not incorporated into cytoophidia during drug treatment. During the preparation of this manuscript two groups presented similar data regarding the regulation of CTPsyn enzymatic activity by filament formation (Barry et al., 2014; Noree et al., 2014). Both studies broadly agree with the assertion that CTPsyn activity is downregulated by incorporation of monomers into cytoplasmic filaments through polymerisation. However, some controversy exists regarding the incorporation of multimers into the filament structure. Barry et al. have shown that the tetramer form of CTPsyn is incorporated into cytoplasmic filaments, whereas Noree et al. have suggested that dimers are the constituent components. This discrepancy could be explained by lack of conservation of filament forming properties in the model organisms studied. Our data provides some support for the fact that dimers are incorporated into cytoophidia. This discrepancy may be explained by a lack of conservation between prokaryotic and eukaryotic CTPsyn, as Barry et al. have focused on bacteria whereas Noree et al. and this manuscript have described yeast and metazoan models respectively. However, this explanation is unsatisfying due to the high sequence identity and conservation of function of CTPsyn. Further investigation into the assembly of eukaryotic cytoophidia will be required to resolve this issue.

## Supplementary Material

Supplementary Material
