# Supplementary material for: Nucleotide synthesis is regulated by cytoophidium formation during neurodevelopment and adaptive metabolism
Source: Biol Open. 2014 Oct 17;3(11):1045–56. doi: 10.1242/bio.201410165 (PMC4232762; doi:10.1242/bio.201410165)
Supplement: Supplementary Material [file supp_3_11_1045__index.html]

Nucleotide synthesis is regulated by cytoophidium formation during neurodevelopment and adaptive metabolism — Supplementary Material 

# Nucleotide synthesis is regulated by cytoophidium formation during neurodevelopment and adaptive metabolism

## bio.201410165 Supplementary Material

**Files in this Data Supplement:**

- Supplementary Material - Gabriel N. Aughey et al. doi: 10.1242/bio.201410165
