# Supplementary material for: Nucleotide synthesis is regulated by cytoophidium formation during neurodevelopment and adaptive metabolism
Source: Biol Open. 2014 Oct 17;3(11):1045–56. doi: 10.1242/bio.201410165 (PMC4232762; doi:10.1242/bio.201410165)
Supplement: Supplementary Material [file supp_bio.201410165_bio.201410165-s1.pdf]

Biology Open (2014) 000, 1–12 doi:10.1242/bio.201410165

Supplementary Material

Gabriel N. Aughey et al. doi: 10.1242/bio.201410165

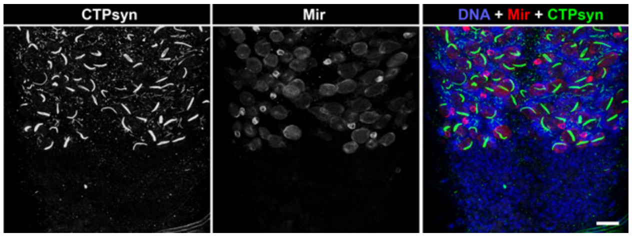

Fig. S1. Overexpression of *CTPsyn* in neuroblasts. *CTPsyn* was overexpressed using *Pros-GAL4* which expresses in neuroblasts, neurons, and glia. Miranda staining shows the location of the neuroblasts and ganglion mother cells (neuroblast daughters). Overexpression of *CTPsyn* in neuroblasts does not result in any overt phenotype. These images are representative of all 10 animals studied at L3. Scale bar: 10 μm.

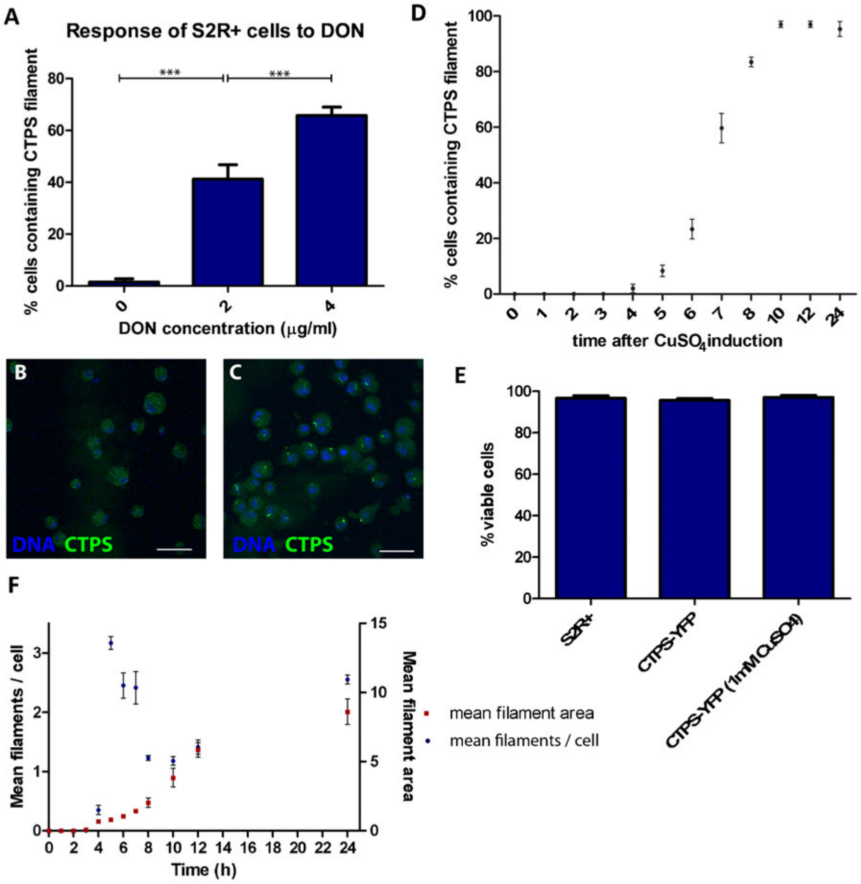

Fig. S2. Inhibition or overexpression of CTPsyn in *Drosophila* cells. (A) *Drosophila* S2R+ cells display similar filament forming characteristics to previously studied human cell lines. Cells were incubated with 0, 2 or 4 4 μg/ml DON for 16 hours. (B) Untreated cells show very low incidence of CTPsyn filaments. (C) Treatment of S2R+ cells with 4 μg/ml DON increases CTPsyn filament formation. 100 cells from three individual wells were counted. An average of three independent experiments is shown. Error bars show SEM. One way ANOVA, ***P≤0.001. Scale bars: 20 μm.
(D) Characterisation of CTPsyn-YFP cell line. Percentage of cells containing at least one CTPsyn filament after $CuSO_4$ induction over 24 hours. No YFP expression was visible before 4 hours. Filaments can be induced to form in the majority of cells at 8 hours post induction. (E) Cell viability measured by trypan blue dye exclusion assay 8 hours after addition of $CuSO_4$. Expression of CTPsyn-YFP transgene caused no significant change in cell viability compared to no $CuSO_4$ treatment or wild-type S2R+ controls.
(F) Measurement of mean number of filaments visible per cell and mean filament area (arbitrary units) at various times after $CuSO_4$ induction. Mean filament area increases with time. Mean number of filaments increases rapidly after 4 hours, although the mean size is small. After 8 hours between one and two large filaments are present per cell. All measurements were taken from a minimum of five sites per well (at least 50 cells) across three replicates. Error bars show SEM.

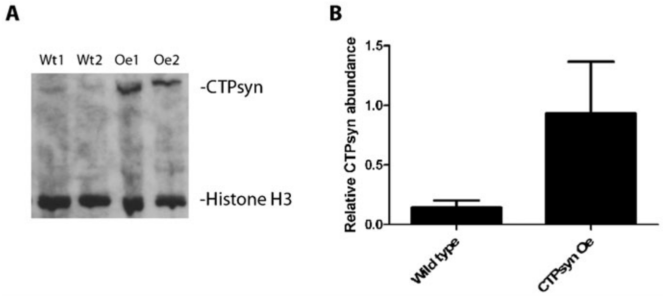

Fig. S3. ***CTPsyn*** **overexpression in** ***Drosophila.*** (A) Western blot indicates that CTPsyn protein levels are much higher in overexpression flies than wild-type controls. (B) Quantification of CTPsyn band intensity in panel A normalised to histone H3 indicates that CTPsyn protein levels are around five-fold higher in *CTPsyn* overexpressing flies.

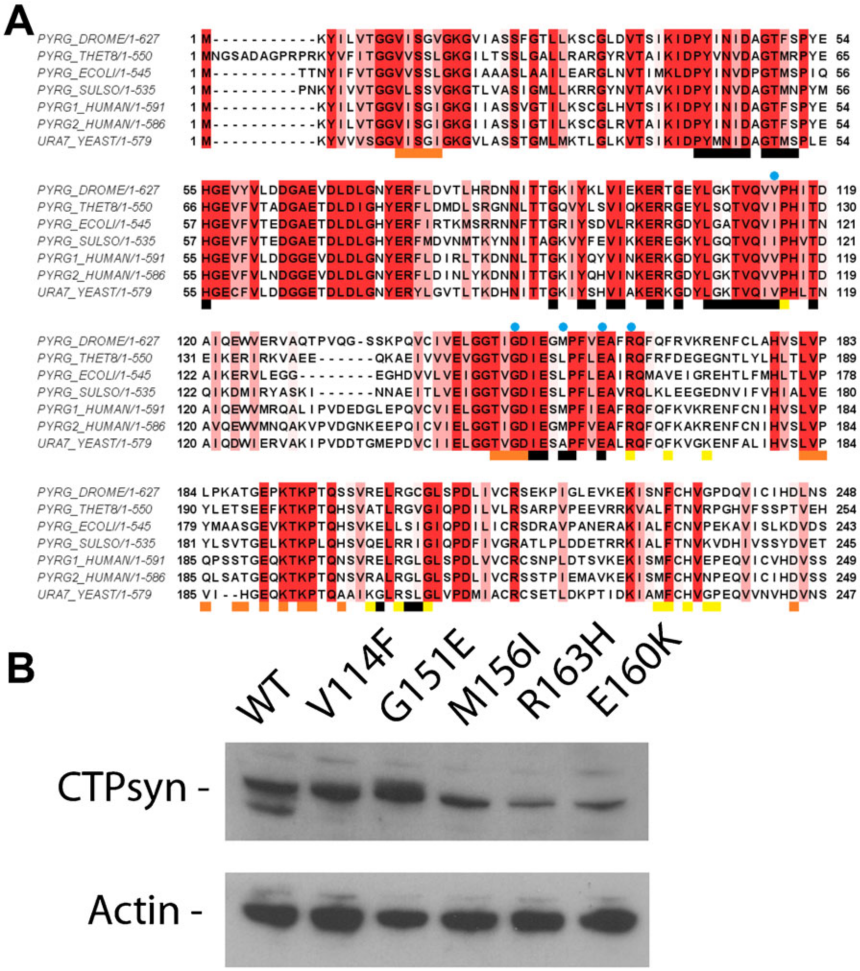

Fig. S4. Point mutations in ***CTPsyn.*** (A) Sequence alignment of CTPsyn orthologues, including *Drosophila* (cytoophidium-forming isoform), *T. thermophilus*, *E. coli*, *S. solfataricus*, human *CTPsyn1*, human *CTPsyn2* and *S. cerevisiae*. Black bars indicate monomer–monomer interfaces, orange bars indicate one dimer–dimer (tetramer) interface, yellow bars indicate the other tetramer interface (data from Lauritsen et al., 2011). Identical residues in the alignment are marked by red boxes; similar residues are shown in lighter red. Mutants described in this manuscript are indicated by blue circles. (B) Western blot data showing relative CTPsyn abundance in cells transfected with mutant *CTPsyn* expression constructs. $CTPsyn^{R163H}$ and $CTPsyn^{E160K}$ appear to have significantly decreased protein levels compared to wild-type, whilst CTPsyn in the remaining mutants is comparable.

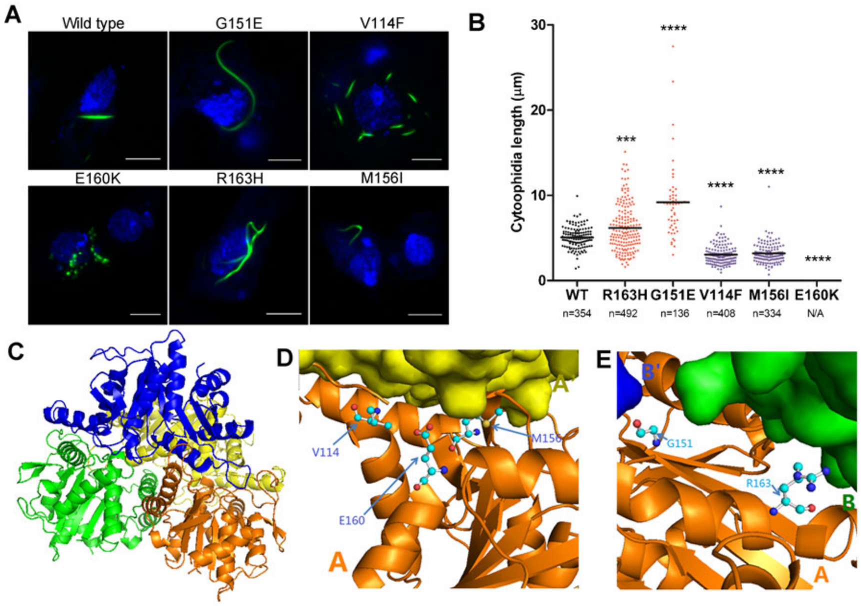

Fig. S5. Mutations at the CTPsyn oligomer interface disrupt cytoophidia assembly. (A) *CTPsyn*^{E160K} substitution completely disrupts cytoophidium formation when ectopically overexpressed in S2R+ cells, although punctate CTPsyn structures are still visible in the cytoplasm. Mutations modifying residues at the dimer–dimer interface: *CTPsyn*^{G151E} and *CTPsyn*^{R163H} (predicted to disrupt CTPsyn tetramer formation), resulted in significantly longer cytoophidia compared to wild type CTPsyn. Mutations modifying residues at the monomer–monomer interface: *CTPsyn*^{V114F} and *CTPsyn*^{M156I} (predicted to disrupt CTPsyn dimer formation), resulted in significantly shorter cytoophidia compared to wild type. Scale bars: 20 µm. Images are representative. (B) Quantification of cytoophidium lengths. At least 50 cells were counted per well. One way ANOVA. ***$P<0.001$, **$P<0.01$, *$P<0.05$. (C) Representation of *Drosophila* CTPsyn tetramer. The CTPsyn tetramer consists of a pair of dimers, shown in yellow/orange and green/blue. (D) Mutagenesis sites on dimer interface of A (orange)–A′ (yellow). A′ is shown as surface view, mutated residues are shown with individual atoms, red: oxygen, dark blue: nitrogen, light blue: carbon, yellow: sulphur. (E) Mutagenesis sites on tetramer interface of A (orange)–B (green), A–B′ (blue). B and B′ are shown as surface view, mutated residues are shown with individual atoms, red: oxygen, dark blue: nitrogen, light blue: carbon. (Picture generated with pyMOL).

Biology Open
